# Red Ginseng Inhibits Tau Aggregation and Promotes Tau Dissociation *In Vitro*

**DOI:** 10.1155/2020/7829842

**Published:** 2020-06-30

**Authors:** Soo Jung Shin, Yong Ho Park, Seong Gak Jeon, Sujin Kim, Yunkwon Nam, Sang-Muk Oh, Yong Yook Lee, Minho Moon

**Affiliations:** ^1^Department of Biochemistry, College of Medicine, Konyang University, Daejeon 35365, Republic of Korea; ^2^The Korean Ginseng Research Institute, Korea Ginseng Corporation, Gajeong-ro 30, Shinseong-dong, Yuseong-gu, Daejeon 34128, Republic of Korea

## Abstract

Tau, a microtubule-associated protein expressed in mature neurons, interacts with tubulin to promote the assembly and stabilization of microtubules. However, abnormally hyperphosphorylated tau dissociates from microtubules and self-aggregates. Tau aggregates, including paired helical filaments and neurofibrillary tangles, promote neuronal dysfunction and death and are the defining neuropathological feature of tauopathies. Therefore, suppressing tau aggregation or stimulating the dissociation of tau aggregates has been proposed as an effective strategy for treating neurodegenerative diseases associated with tau pathology such as Alzheimer's disease (AD) and frontotemporal dementia. Interestingly, ginsenosides extracted from *Panax ginseng* reduced the hippocampal and cortical expression of phosphorylated tau in a rat model of AD. However, no studies have been conducted into the effect of red ginseng (RG) and its components on tau pathology. Here, we evaluated the effect of Korean red ginseng extract (KRGE) and its components on the aggregation and disassociation of tau. Using the thioflavin T assay, we monitored the change in fluorescence produced by the aggregation or disassociation of tau K18, an aggregation-prone fragment of tau_441_ containing the microtubule-binding domain. Our analysis revealed that KRGE not only inhibited tau aggregation but also promoted the dissociation of tau aggregates. In addition, the KRGE fractions, such as saponin, nonsaponin, and nonsaponin fraction with rich polysaccharide, also inhibited tau aggregation and promoted the dissociation of tau aggregates. Our observations suggest that RG could be a potential therapeutic agent for the treatment of neurodegenerative diseases associated with tauopathy.

## 1. Introduction

Tau, a microtubule-associated protein expressed in neurons, interacts with tubulin and promotes the assembly and stabilization of microtubules [1, 2]. Alternative splicing of the *MAPT* (microtubule-associated protein tau) gene produces six isoforms of tau. These are classified according to the number of repeats of 29 amino acids on the N-terminal region (N: zero, one, or two) and the number of microtubule-binding domain repeats (R: three or four) on the C-terminal region [3, 4]. The largest tau isoform is 4R2N tau, and this isoform is the most effective at promoting microtubule assembly [5, 6]. As a microtubule-associated phosphoprotein, the affinity of tau for microtubules is dependent on its phosphorylation level, and normal tau phosphorylation is essential for neuronal plasticity and axonal outgrowth [7, 8]. However, abnormally hyperphosphorylated tau is released from microtubules due to its reduced biological activity and induces synaptic terminal alteration and axonal degeneration, which can result in cognitive impairment [9]. In addition, tau released from microtubules self-assembles into neurotoxic insoluble aggregates such as paired helical filaments, straight filaments, and neurofibrillary tangles (NFTs) [10]. In particular, NFTs in the brain are a histopathological feature of tauopathies such as Alzheimer's disease (AD), frontotemporal dementia, Parkinson's disease, Pick's disease, and progressive supranuclear palsy [11–15].

Abnormally hyperphosphorylated tau inhibits and disrupts the assembly of microtubules [16]. In addition, numerous studies have demonstrated the toxicity of abnormal tau aggregates in neurons and glial cells [16]. While soluble tau is nontoxic, tau aggregates promote the degeneration of N2a neuroblastoma cells [17]. Moreover, tau dimers suppress axonal transport in isolated squid axoplasm [18], and the neurotoxicity of tau trimers was demonstrated in both SH-SY5Y cells and the mouse hippocampal neurons [19, 20]. Interestingly, several studies have shown that tau oligomers and aggregates can be anterogradely propagated between cells via exosomes, endocytosis, and macropinocytosis both *in vitro* and *in vivo* [21–24]. Furthermore, insoluble oligomeric tau has been implicated in the dysfunction of the ubiquitin-proteasome system [25]. Moreover, mice expressing antiaggregation mutations in tau do not exhibit tau-related neuropathology [26], and inhibition of tau aggregation alleviates tauopathy in the *C. elegans* model of tauopathy and P301S tau transgenic mice [27, 28]. Indeed, clinical trials are currently underway to investigate the efficacy of methylene blue (Texas Alzheimer's Research and Care Consortium), NPT088 (Proclara), and LY3303560 (Lilly), all of which are agents that that can inhibit, dissociate, and neutralize tau aggregation, for the treatment of AD [29]. Thus, inhibition of tau aggregation is a well-established therapeutic strategy for the treatment of tauopathies including AD [30].

Ginseng, the root of *Panax ginseng* Meyer, is a representative medicinal herb in East Asian countries. Ginseng contains various bioactive components such as ginsenosides, flavonoids, polyphenols, and polysaccharides [31]. Interestingly, ginseng can be processed into red ginseng (RG) through a series of steam and drying processes to enhance the pharmacological efficacy of the bioactive substances present in ginseng [32]. In addition, ginseng has been used as a medicinal herb that can treat various diseases such as cancer, diabetes, and cardiovascular diseases [33–35]. Moreover, RG has been reported to mitigate AD-related pathology in animal models of AD and patients with AD [36–40]. Furthermore, ginsenosides have been shown to ameliorate AD-related pathology, potentially by modulating amyloid-*β* (A*β*) processing, phosphorylated tau (p-tau) expression, and choline acetyltransferase expression [41, 42]. Interestingly, although the mechanism underlying their effect remains unknown, treatment with ginsenosides extracted from *Panax ginseng* reduced p-tau deposition in a rat model of AD [42]. However, to date, the effects of RG and its components on the aggregation and dissociation of tau have not yet been examined. In this study, we investigated the effect of Korean red ginseng extract (KRGE) and KRGE fractions, including the saponin fraction (SF), nonsaponin fraction (NSF), and nonsaponin fraction with rich polysaccharide (NFP), on tau aggregation and dissociation. To do so, we performed the thioflavin T (ThT) assay with tau K18 fragment, a microtubule-binding domain repeat of the 4R2N tau isoform.

## 2. Materials and Methods

### 2.1. Chemicals and Materials

Commercial KRGE was purchased from the Korea Ginseng Corporation (Cheong-Kwan-Jang, Daejeon, Republic of Korea). KRGE was prepared from 6-year-old Korean *Panax ginseng* Meyer. Thioflavin T (Basic Yellow 1, ThT) and Dulbecco's phosphate-buffered saline (PBS) were purchased from the Tokyo Chemical Industry Co., Ltd. (Tokyo, Japan; T0558) and Welgene Inc. (Gyeongsan, Republic of Korea; LB 001-02), respectively. Methylene blue (M9140-25G), 1,4-dithiothreitol (DTT, D0632-5G), and heparin sodium salt from porcine intestinal mucosa (H3393-25KU) were purchased from Sigma-Aldrich (St. Louis, MO, USA). Some carbohydrate-digesting enzymes, namely, *α*-amylase from *Aspergillus oryzae*, amyloglucosidase from *Aspergillus niger*, and pectinesterase from orange peel, were all obtained from Sigma-Aldrich, whereas polygalacturonase from *Aspergillus aculeatus* was purchased from Megazyme (Bray, Ireland). A*β*_1-42_ peptide was purchased from Bachem, Inc. (Torrance, CA, USA; H-1366).

### 2.2. Extraction of KRGE Fractions

In the previous study, the commercial KRGE was freeze-dried and ground into a fine powder [36]. The KRGE for SF, NSF, and NFP (Figure S1) was supplied by the Korea Ginseng Corporation (Buyeo, Republic of Korea) according to the reported method [32]. The SF and NSF were prepared from the KRGE by adsorption chromatography with Diaion HP20 (Mitsubishi Chemical Industries, Ltd.). The KRGE was diluted to 10% in water and then filtered. The diluted KRGE was subjected to adsorption chromatography using Diaion HP20 and was then sequentially eluted with water, 30% EtOH, and 95% EtOH. The first two fractions (H_2_O and 30% EtOH) were combined, concentrated, and spray-dried to produce the NSF. The last fraction (95% EtOH) was concentrated and spray-dried to produce the SF. The extraction procedure for the NFP from KRGE was carried out via EtOH precipitation, enzyme hydrolysis, and size exclusion chromatography as previously described [43]. Briefly, 95% EtOH was added to the KRGE for precipitation. The solution was treated with *α*-amylase and amyloglucosidase, and the enzyme reaction was then quenched. Following this, 4 volumes of 80% EtOH were then added to precipitate the polysaccharide-rich fraction. The polysaccharide-rich fraction was treated with pectinesterase and hydrolyzed with polygalacturonase. The lysates were eventually prepared as the dried NFP after lyophilization.

### 2.3. Characterization of KRGE Fractions

The ingredients of KRGE powder were determined as follows: ginsenoside and acidic polysaccharide (AP) content [36]. The analyses of ginsenoside [44] and AP [45] were based on the respective method. The analysis of all the contents was performed as previously described [36]. Briefly, to analyze ginsenosides, the samples was extracted with methanol in an ultrasonicator and then subjected to centrifugal separation. The final filtered samples were injected into a Waters ACQUITY UPLC system (Waters, Milford, MA, USA). Eleven ginsenosides were detected using a UPLC Photodiode Array system (Waters). To analyze the AP content, we used the carbazole-sulfuric acid method. The ginsenoside and AP content of KRGE and its fractions are shown in Table 1.

### 2.4. Expression and Purification of Recombinant Tau K18

In this study, we used a recombinant K18 fragment which contains four repeats of a critical binding domain associated with the aggregation of tau which thus aggregates faster than the full-length tau [46, 47]. The tau K18 fragment (129 amino acids), which was cloned from full-length human tau (hTau40), was expressed in and purified from *Escherichia coli* BL21 (DE3) cells. Tau K18 was commercially purified by BioProgen (Daejeon, Republic of Korea). The amino acid sequence of tau K18 is as follows: QTAPVPMPDL KNVKSKIGST ENLKHQPGGG KVQIINKKLD LSNVQSKCGS KDNIKHVPGG GSVQIVYKPV DLSKVTSKCG SLGNIHHKPG GGQVEVKSEK LDFKDRVQSK IGSLDNITHV PGGGNKKIE. The DNA sequence of tau K18 was obtained using codon optimization performed by Bioneer (Figure S2). The purity of the proteins was analyzed by 20% sodium dodecyl sulfate-polyacrylamide gel electrophoresis (Figure S3).

### 2.5. Evaluation of the Effect of KRGE and Its Fractions on Tau K18 Aggregation

The ThT assay was performed to investigate the effect of KRGE and its fractions on tau aggregation. ThT is known to bind *β*-sheet-rich protein aggregates and produce fluorescence [48]. To induce tau aggregation, synthetic tau K18 was dissolved in PBS (pH 7.4). Polymerization of the tau protein was induced by preparing in a 1 mL solution containing PBS (pH 7.4), 1 mg/mL tau K18, 0.1 mg/mL heparin, and 100 *μ*M DTT. Various concentrations of KRGE (10, 50, 100, 250, and 500 *μ*g/mL), methylene blue solution (positive control), and KRGE fractions (SF, NSF, and NFP; 10-500 *μ*g/mL) were incubated with the aggregation mixture for 21 hours (Figure S4). After the incubation period, ThT solution (15 *μ*M in 50 mM glycine buffer, pH 8.9) was added to a 96-well plate containing samples and the aggregation mixture, and the mixture was incubated for 3 hours. The fluorescence intensity of ThT was measured at excitation and emission wavelengths of 440 and 484 nm with a SpectraMax iD3 Multi-Mode Microplate Reader (Molecular Devices, San Jose, CA, USA).

The Tecnai G2 spirit TWIN transmission electron microscope (TEM) (Field Electron and Ion Company, Hillsboro, OR, USA) was used for the imaging of tau K18 aggregation. Tau K18 (1 mg/mL) in PBS (pH 7.4) was incubated with 0.1 mg/mL heparin and 100 *μ*M DTT at 37°C for 24 hours with the 500 *μ*g/mL KRGE, SF, NSF, or NFP. The tau K18 sample was placed on a 400-mesh collodion-carbon-coated copper grid (Veco B.V., Eerbeek, Netherlands) and adsorbed for 1 min. Then, the grid was stained with 2.5% uranyl acetate for 1 min, and excess liquid at the border of the grid was removed by touching it with filter paper. Tau K18 images were captured at 120 kV. Length of the tau K18 aggregates was quantified by ImageJ software (NIH, Bethesda, MD, USA).

### 2.6. Evaluation of the Effect of KRGE and Its Fractions on the Dissociation of Tau K18 Aggregates

The ThT assay was performed to investigate the effect of KRGE and its fractions on the dissociation of preformed tau aggregates. To induce tau aggregation, purified K18 (1 mg/mL) in phosphate-buffered saline (pH 7.4) was incubated with 0.1 mg/mL heparin and 100 *μ*M DTT at 37°C for 24 hours (Figure S4). KRGE and KRGE fractions, including the SF, NSF, and NFP, were added to the aggregation mixture after 21 hours of incubation. At the end of the incubation period, ThT (15 *μ*M in 50 mM glycine buffer, pH 8.9) was added to a 96-well plate and incubated for 3 hours. The fluorescence intensity of tau-bound ThT was measured at Ex/Em = 440 nm/484 nm with a SpectraMax iD3 Multi-Mode Microplate Reader (Molecular Devices).

### 2.7. Evaluation of the Effect of KRGE on A*β*_1-42_ Aggregation

The ThT assay was performed to investigate the effect of KRGE on A*β*_1-42_ aggregation. To induce A*β*_1-42_ aggregation, synthetic A*β* was diluted in distilled water to a final concentration of 25 *μ*M. Various concentrations of KRGE (100, 250, and 500 *μ*g/mL) and morin (positive control; 100 *μ*M) [49–51] were incubated with A*β*_1-42_ peptide solution for 3 hours at 37°C. After incubation, ThT solution (15 *μ*M in 50 mM glycine buffer, pH 8.9) was added to a 96-well plate and incubated for 3 hours. The fluorescence intensity of ThT was measured at Ex/Em = 440 nm/484 nm with a SpectraMax iD3 Multi-Mode Microplate Reader (Molecular Devices).

### 2.8. Statistical Analysis

All statistical analyses were conducted using GraphPad Prism 7.0 software (GraphPad Software, Inc., La Jolla, CA, USA). Data are presented as mean ± standard error of the mean. The Kolmogorov-Smirnov test was used to evaluate the normality of the data. When the Kolmogorov-Smirnov test showed no significant differences, Levene's test was performed for analysis of variance between groups. To compare data between three or more groups, one-way analysis of variance followed by Tukey's post hoc test was used. Data which were not normally distributed were analyzed by the Kruskal-Wallis test followed by Dunn's post hoc test. A *p* value < 0.05 indicated statistical significance. IC_50_ and DC_50_ values were determined by nonlinear regression analysis.

## 3. Results

### 3.1. Characterization of KRGE and KRGE Fractions

According to the previous results of the component analysis of KRGE powder, total ginsenoside content was 24.56 mg/g and AP content was 98.46 mg/g [36]. In the present study, the total ginsenoside content of the SF was almost 14.5 times (356.37 mg/g) that of the KRGE powder (24.56 mg/g) (Table 1). No ginsenosides were detected in the NSF and NFP. The NFP showed an 86-fold higher AP content than the SF (438.08 mg/g and 5.09 mg/g, respectively). KRGE powder (98.46 mg/g) and the NSF (98.02 mg/g) had similar AP content. The composition of all samples is summarized in Table 1.

### 3.2. KRGE Inhibits Tau Aggregation and Induces Tau Dissociation

**Figure 1:**
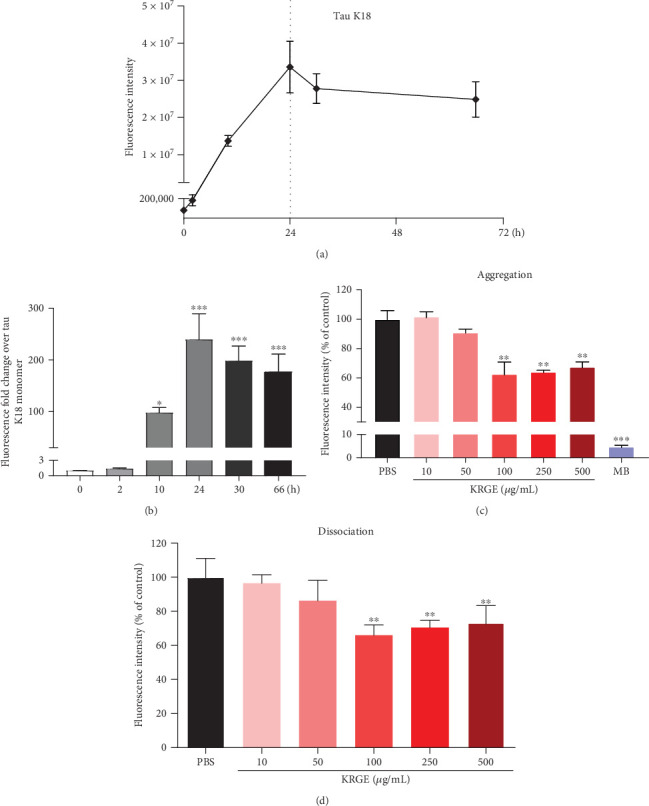
The effect of Korean red ginseng extract (KRGE) on the aggregation and dissociation of tau K18. (a) The aggregation of tau K18 fragments was monitored using the thioflavin T (ThT) assay for 72 hours. (b) The fold changes of the aggregated tau were analyzed by assessing the fluorescence intensity of ThT and normalized to tau K18 monomer. (c) The fluorescence of ThT in the presence and absence of KRGE and methylene blue (MB; 100 *μ*M). (d) The dissociation of preformed tau aggregates was analyzed by assessing the fluorescence intensity of ThT in the presence and absence of KRGE. Values represent the mean ± standard error of the mean of three independent experiments. Statistical significance was determined by one-way analysis of variance followed by Tukey's multiple comparison test. ^∗^*p* < 0.05; ^∗∗^*p* < 0.01; ^∗∗∗^*p* < 0.001.

We investigated the effect of KRGE on tau aggregation and disassociation using the ThT assay (Figure S4). Prior to treatment with KRGE, tau K18 fragments were incubated with heparin for 72 hours to monitor alterations in ThT fluorescence intensity according to the aggregation of tau K18 (Figure 1(a)). In addition, the fold change was analyzed to confirm the aggregated tau for the tau monomer over time (Figure 1(b)). After incubating tau K18 for 24 hours, the highest intensity was observed by 239.4-folds. Next, tau K18 fragments were incubated with heparin in the presence or absence of KRGE. We observed that KRGE (10, 50, 100, 250, and 500 *μ*g/mL) significantly inhibited tau aggregation (Figure 1(c)) Since methylene blue is known to hinder the aggregation of tau [52], we used it as a positive control. In addition, the intensity of ThT fluorescence was significantly reduced when KRGE (10, 50, 100, 250, and 500 *μ*g/mL) was incubated with preformed aggregates of tau K18 (Figure 1(d)). However, KRGE treatment did not affect the aggregation of monomeric A*β*_1-42_ (Figure S5). These results indicate that KRGE can modulate tau aggregation and disassociation.

### 3.3. Fractions of KRGE Interfere with Tau K18 Aggregation

**Figure 2:**
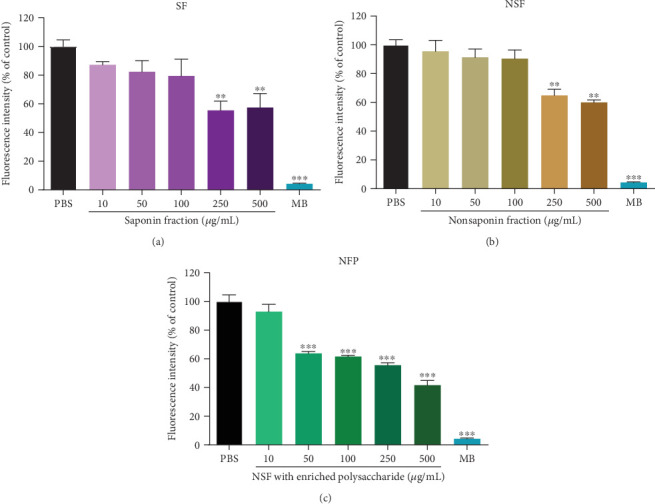
The inhibitory effect of Korean red ginseng extract (KRGE) fractions on tau K18 aggregation. Tau aggregation was analyzed by assessing the fluorescence intensity of thioflavin T (ThT) in the presence and absence of (a) the saponin fraction (SF), (b) the nonsaponin fraction (NSF), and (c) the NSF with rich polysaccharide (NFP). Methylene blue (MB; 100 *μ*M), a known inhibitor of tau aggregation, was used as a positive control. Values represent the mean ± standard error of the mean of three independent experiments. Statistical significance was determined by one-way analysis of variance followed by Tukey's multiple comparison test. ^∗∗^*p* < 0.01; ^∗∗∗^*p* < 0.001.

**Figure 3:**
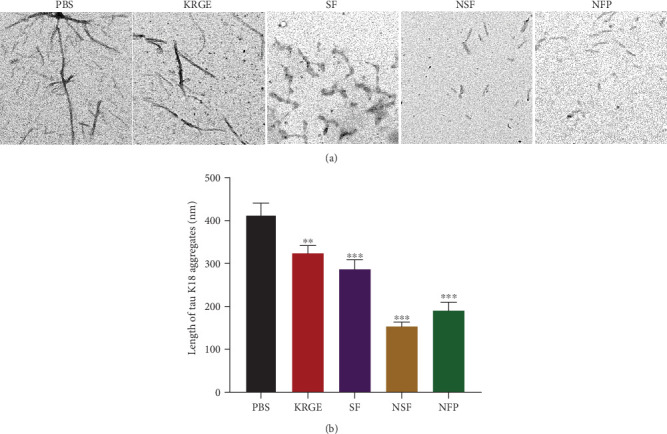
The effect of KRGE and KRGE fractions on the aggregation of tau K18 using the transmission electron microscopy (TEM). (a) Images of tau K18 aggregates in the absence or presence of 500 *μ*g/mL KRGE or KRGE fractions (scale bar = 200 nm). (b) The length of the tau K18 aggregates was quantified in TEM images. Values represent the mean ± standard error of the mean. Statistical significance was determined by one-way analysis of variance followed by Tukey's multiple comparison test. ^∗∗^*p* < 0.01; ^∗∗∗^*p* < 0.001.

Next, we examined which fractions of KRGE have a direct effect on tau aggregation using the ThT assay. The SF of KRGE (250 and 500 *μ*g/mL) significantly inhibited the aggregation of tau K18 (Figure 2(a)). In addition, incubation with the NSF (250 and 500 *μ*g/mL) also significantly inhibited the aggregation of tau (Figure 2(b)). Moreover, treatment with the NFP inhibited tau aggregation in a concentration-dependent manner (Figure 2(c)). Methylene blue was used as a positive control and significantly reduced tau aggregation. To confirm the inhibitory effect of KRGE fractions on tau K18 aggregation, tau K18 aggregates were imaged with a TEM and the length of the aggregates was quantified (Figure 3(a)). Significant reduction in the length of tau K18 aggregates was observed after treatment with KRGE or KRGE fractions at a dose of 500 *μ*g/mL (Figure 3(b)). There was no significant difference in length of tau K18 aggregates between NSF and NFP. Taken together, we demonstrated that KRGE fractions can effectively prevent tau aggregation.

### 3.4. KRGE Fractions Dissociate Preformed Tau K18 Aggregates

**Figure 4:**
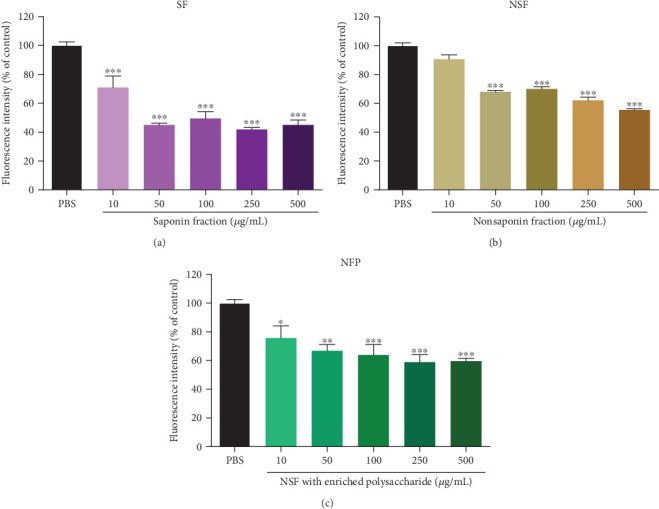
The stimulatory effect of Korean red ginseng extract (KRGE) fractions on tau K18 dissociation. Tau dissociation was analyzed by assessing the fluorescence intensity of thioflavin T (ThT) in the presence and absence of (a) the saponin fraction (SF), (b) the nonsaponin fraction (NSF), and (c) the NSF with rich polysaccharide (NFP). Values represent the mean ± standard error of the mean of three independent experiments. Statistical significance was determined by one-way analysis of variance followed by Tukey's multiple comparison test. ^∗^*p* < 0.05; ^∗∗^*p* < 0.01; ^∗∗∗^*p* < 0.001.

Furthermore, we examined whether KRGE fractions can promote the disassociation of tau aggregates using the ThT assay. Tau K18 fragments were incubated with heparin for 24 hours, and preformed tau aggregates were then incubated with KRGE fractions (SF, NSF, and NFP) for 21 hours (Figure S4). ThT fluorescence was significantly reduced by incubation of the SF with tau K18 fragments (Figure 4(a)). Additionally, we found that incubation of tau K18 fragments with the NSF caused a significant decrease in the fluorescence intensity of ThT (Figure 4(b)). The ThT assay revealed that the NFP also stimulated tau dissociation, as demonstrated by the observed decrease in ThT fluorescence (Figure 4(c)). Overall, our data showed that KRGE fractions are able to disassociate preformed tau fibrils.

## 4. Discussion

**Figure 5:**
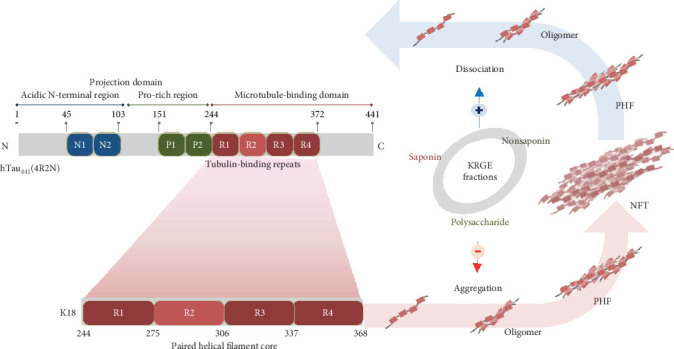
A schematic illustration of the structure of tau K18 and the modulatory effects of KRGE fractions on tau K18 aggregation/dissociation. KRGE and its constituents exhibited modulatory effects on tau aggregation and dissociation. KRGE: Korean red ginseng extract; NFT: neurofibrillary tangle; PHF: paired helical filament.

Ginseng is known to have beneficial effects in neurodegenerative diseases involving the pathological aggregation of tau in the brain [53]. Among the various types of processed ginseng, RG has been reported to improve cognitive function in both rodents and humans [54–56]. In particular, the antidementia effect of RG has been demonstrated in animal models of AD and AD patients [36, 37, 39, 40]. Therefore, the effects of ginsenosides, the main active components of RG, on AD-related pathology such as neuroinflammation [57, 58], mitochondrial dysfunction [59–62], and amyloidogenic mechanism [63–66] have previously been studied. However, to the best of our knowledge, only one study has examined the effect of ginsenosides on tau pathology [42]. Moreover, although polysaccharides and nonsaponin compounds are the main constituents of RG, their effect on tau pathology has not yet been studied. To investigate whether RG can alleviate tau pathology, we evaluated the effect of KRGE and its components on tau aggregation and dissociation using tau K18 fragments and ThT fluorescence. Tau K18 fragments comprise four tubulin-binding repeats and make up the paired helical filament core in the microtubule-binding domain of full-length hTau_441_ (4R2N) (Figure 5). These fragments have been used in numerous studies to characterize tau aggregation and dissociation *in vitro* [46, 67–71].

Remarkably, our results demonstrated that treatment with KRGE can modulate the aggregation and dissociation of tau K18 fragments (Figures 1–4). Furthermore, to determine which components of KRGE contribute to its antiaggregation effect, we evaluated the effect of KRGE fractions, including the SF, NSF, and NFP, on the aggregation of tau K18 fragments. All KRGE fractions inhibited the aggregation of tau K18 fragments at high concentrations (250 and 500 *μ*g/mL). Among the KRGE fractions, the NFP most potently inhibited tau aggregation (Figure 2). Given the proportion of polysaccharides in each of the KRGE fractions (Table 1), the antiaggregation effect of KRGE on tau appears to primarily derive from the NFP. Moreover, we investigated the effect of KRGE fractions on the disassociation of tau K18 aggregates. The SF most effectively induced the dissociation of tau K18 fragments, and the NFP also promoted tau dissociation even at the lowest concentration studied (10 *μ*g/mL) (Figure 4). Based on these results, we derived the IC_50_ and DC_50_ of KRGE extract on the aggregation and dissociation of tau K18 (Table 2). These results suggest that KRGE fractions may modulate the aggregation and dissociation of tau via different mechanisms.

**Figure 6:**
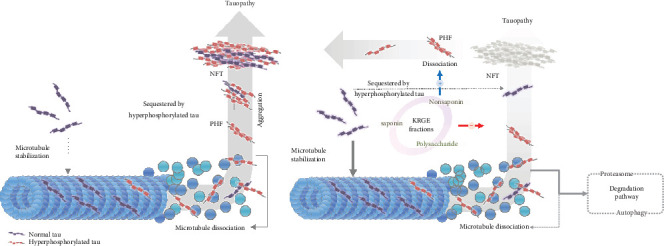
Tau pathomechanisms and proposed mechanism underlying the modulatory effect of KRGE and its fractions on tau K18 aggregation/dissociation. KRGE: Korean red ginseng extract; NFT: neurofibrillary tangle; PHF: paired helical filament.

The toxicity of tau aggregates is controversial and is thought to depend on the form of aggregate and the microenvironment [72]. In a previous study, mice with human tau mutations exhibited behavioral deficits, neuronal loss, and progressive NFT deposition with age. Reducing the expression of mutant tau ameliorated tau-related pathological symptoms such as neuronal loss and memory, but NFTs continued to accumulate in the brain [73]. These findings suggest that neurodegeneration and cognitive dysfunction associated with tauopathy are not NFT-dependent. Rather, soluble tau aggregates such as oligomers may directly contribute to the progression of tau-related pathology. However, as mentioned above, many studies have demonstrated that inhibiting tau aggregation or promoting tau dissociation may also contribute to alleviating tauopathy. Despite this controversy, targeting tau aggregates is a therapeutic strategy that accounts for a large proportion of clinical trials for the treatment of AD [29]. In addition, a number of studies demonstrated that inhibiting tau aggregation or promoting the dissociation of tau aggregates can have a beneficial effect through various mechanisms such as propagation, degradation, and sequestration (Figure 6). Self-aggregating hyperphosphorylated tau has been reported to sequester normal tau during the aggregation process [74–76]. Therefore, inhibiting tau aggregation may reduce normal tau sequestration by hyperphosphorylated tau and contribute to microtubule stabilization. Furthermore, because abnormal tau aggregates are known to undergo autophagic and proteasomal degradation [77–79], dissociating insoluble forms of tau aggregates, such as NFTs or paired helical filaments, into smaller soluble forms may contribute to the removal of abnormally phosphorylated tau. Moreover, inhibiting tau aggregation and promoting the dissociation of tau aggregates may suppress intercellular tau propagation, which is known to induce neuroinflammation by activating astrocytes and microglia [80, 81]. These studies suggest that inhibiting tau aggregation or promoting tau dissociation may be promising strategies for the treatment of tauopathies (Figure 6).

In our previous study, administration of KRGE reduced A*β* accumulation in the brains of A*β*-overexpressing transgenic mice [36]. Thus, we analyzed the effect of KRGE on A*β* aggregation through ThT fluorescence to determine whether KRGE alleviated A*β* accumulation by directly interacting with the A*β* peptide (Figure S5). KRGE did not directly affect the aggregation of A*β*_1-42_, indicating that the reduction of A*β* accumulation shown in the brains of A*β*-overexpressing transgenic mice may not be mediated through direct inhibition of A*β* accumulation, but through alternative pathways, including inhibition of mitochondrial dysfunction, modulation of the A*β* production pathway, or activation of A*β* degradation/clearance.

In future work, we plan to evaluate the *in vivo* efficacy of KRGE and its constituents in animal models that exhibit tau-related pathology such as the triple-transgenic mouse model of AD, which exhibits both A*β* and tau pathology, and P301S tau transgenic mice (a human tauopathy model). Furthermore, we intend to conduct a future study in order to further characterize KRGE fractions and identify the principal components which modulate the aggregation and dissociation of tau.

## 5. Conclusions

Our findings demonstrate that KRGE and its constituents can regulate the aggregation and dissociation of tau and thus may be beneficial for the treatment of neurodegenerative diseases associated with tauopathy.

## Figures and Tables

**Table 1:** Characterization of Korean red ginseng root extract (KRGE) fractions.

		KRGE	SF	NSF	NFP
Ginsenoside (mg/g)	Rb1	6.23	101.79	ND	ND
	Rb2	2.45	35.58	ND	ND
	Rc	2.94	41.74	ND	ND
	Rd	1.27	14.05	ND	ND
	Re	0.93	23.13	ND	ND
	Rf	1.37	20.54	ND	ND
	Rg1	0.64	17.28	ND	ND
	Rg2s	1.78	20.61	ND	ND
	Rg3r	1.77	21.80	ND	ND
	Rg3s	3.50	44.53	ND	ND
	Rh1	1.68	15.33	ND	ND
	Total	24.56	356.37	0.00	0.00
AP (mg/g)		98.46	5.09	98.02	438.08

AP: acidic polysaccharide; ND: not detected; NSF: nonsaponin fraction; NFP: nonsaponin fraction with rich polysaccharide; SF: saponin fraction.

**Table 2:** Determination of IC_50_ and DC_50_ values of the Korean red ginseng root extract (KRGE) for aggregation and dissociation of tau K18.

	Aggregation	Dissociation
	IC_50_ (*μ*g/mL)	DC_50_ (*μ*g/mL)
KRGE	545.0 ± 124.4	713.5 ± 158.3
SF	395.1 ± 89.02	100.2 ± 28.87
NSF	645.4 ± 137.7	370.3 ± 58.65
NFP	179.3 ± 25.51	271.8 ± 69.44

NSF: nonsaponin fraction; NFP: nonsaponin fraction with rich polysaccharide; SF: saponin fraction.

## Data Availability

The data used to support the findings of this study are available from the corresponding author upon request.

## Supplementary Materials

Supplementary Materials Supplementary Figure 1: composition of Korean red ginseng extract: saponin fraction, nonsaponin fraction, and nonsaponin fraction (NSF) with rich polysaccharide. Supplementary Figure 2: amino acid and DNA sequences for synthesis of recombinant tau K18 fragments. Supplementary Figure 3: purification of recombinant tau K18. The recombinant tau K18 was confirmed by sodium dodecyl sulfate-polyacrylamide gel electrophoresis. Marker indicates the molecular weight of the protein, and the rest of the lanes contain purified protein. Supplementary Figuer 4: overview of the thioflavin T (ThT) assay for evaluating the effect of Korean red ginseng extract (KRGE) and KRGE fractions (KRGFs) on the aggregation and dissociation of tau K18 fragments. Supplementary Figure 5: evaluation of the effect of Korean red ginseng extract (KRGE) on A*β*_1-42_ aggregation. (a) Fluorescence intensity during the thioflavin T (ThT) assay. Monomeric A*β*_1-42_ was incubated for 5 hours to allow for oligomerization. (b) The ThT assay was performed to examine the effect of KRGE on A*β*_1-42_ aggregation. When A*β*_1-42_ was incubated in the presence of morin, a well-known inhibitor of A*β* aggregation, A*β*_1-42_ aggregation was dramatically reduced. However, no significant change in A*β*_1-42_ aggregation was observed after incubation with KRGE. Values represent the mean ± standard error of the mean of three independent experiments. Statistical significance was determined by one-way analysis of variance followed by Tukey's multiple comparison test. ^∗∗∗^*p* < 0.001.
