# Supplementary material for: Red Ginseng Inhibits Tau Aggregation and Promotes Tau Dissociation In Vitro
Source: Oxid Med Cell Longev. 2020 Jun 30;2020:7829842. doi: 10.1155/2020/7829842 (PMC7350179; doi:10.1155/2020/7829842)
Supplement: Supplementary Materials — Supplementary Figure 1: composition of Korean red ginseng extract: saponin fraction, nonsaponin fraction, and nonsaponin fraction (NSF) with rich polysaccharide. Supplementary Figure 2: amino acid and DNA sequences for synthesis of recombinant tau K18 fragments. Supplementary Figure 3: purification of recombinant tau K18. The recombinant tau K18 was confirmed by sodium dodecyl sulfate-polyacrylamide gel electrophoresis. Marker indicates the molecular weight of the protein, and the rest of the lanes contain purified protein. Supplementary Figuer 4: overview of the thioflavin T (ThT) assay for evaluating the effect of Korean red ginseng extract (KRGE) and KRGE fractions (KRGFs) on the aggregation and dissociation of tau K18 fragments. Supplementary Figure 5: evaluation of the effect of Korean red ginseng extract (KRGE) on Aβ1-42 aggregation. (a) Fluorescence intensity during the thioflavin T (ThT) assay. Monomeric Aβ1-42 was incubated for 5 hours to allow for oligomerization. (b) The ThT assay was performed to examine the effect of KRGE on Aβ1-42 aggregation. When Aβ1-42 was incubated in the presence of morin, a well-known inhibitor of Aβ aggregation, Aβ1-42 aggregation was dramatically reduced. However, no significant change in Aβ1-42 aggregation was observed after incubation with KRGE. Values represent the mean ± standard error of the mean of three independent experiments. Statistical significance was determined by one-way analysis of variance followed by Tukey's multiple comparison test. ∗∗∗p < 0.001. [file 7829842.f1.pdf]

Supplementary figures

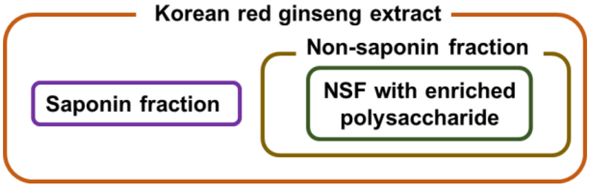

SUPPLEMENTARY FIGURE 1: Composition of Korean red ginseng extract: saponin fraction, non-saponin fraction, and non-saponin fraction (NSF) with rich polysaccharide.

• **Tau K18 fragment (244-372)**

QTAPVPMPDL KNVKSKIGST ENLKHQPGGG KVQIINKKLD LSNVQSKCGS KDNIKHVPGG GSVQIVYKPV DLSKVTSKCG SLGNIHHKPG GGQVEVKSEK LDFKDRVQSK IGSLDNITHV PGGGNKKIE

DNA sequence - Bioneer condon opti.

CAG ACA GCC CCC GTG CCT ATG CCG GAC CTG AAG AAT GTG AAA TCC AAG ATT GGA TCT ACT GAG AAT CTG AAG CAC CAG CCG GGT GGC GGG AAG GTT CAG ATA ATT AAT AAG AAG CTC GAT CTT AGC AAC GTC CAG TCC AAA TGC GGG TCA AAG GAT AAT ATC AAG CAC GTT CCG GGC GGT GGC AGT GTC CAA ATA GTT TAT AAG CCA GTT GAT CTG AGC AAG GTG ACG TCG AAA TGT GGA TCA TTG GGT AAC ATT CAT CAT AAG CCA GGA GGC GGC CAA GTG GAA GTA AAG TCT GAA AAG TTA GAT TTT AAG GAC CGT GTC CAG AGC AAG ATT GGG AGT CTG GAC AAT ATC ACC CAT GTA CCT GGC GGT GGT AAC AAG AAG ATC GAA TAA

SUPPLEMENTARY FIGURE 2: Amino acid and DNA sequences for synthesis of recombinant tau K18 fragments.

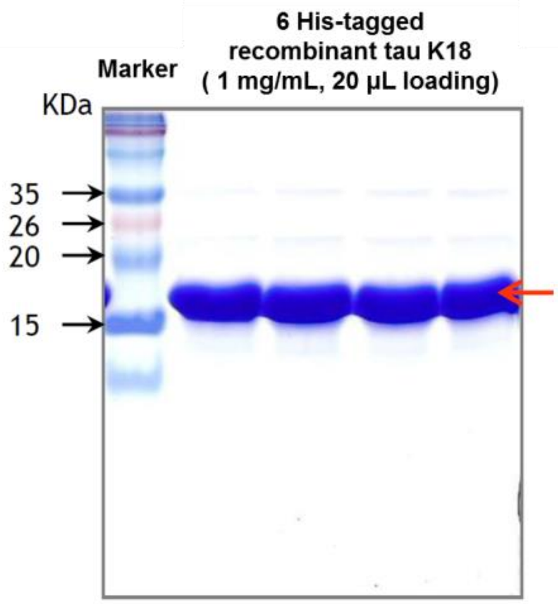

SUPPLEMENTARY FIGURE 3: Purification of recombinant tau K18. The recombinant tau K18 was confirmed by sodium dodecyl sulfate-polyacrylamide gel electrophoresis. Marker indicates the molecular weight of the protein, and the rest of the lanes contain purified protein.

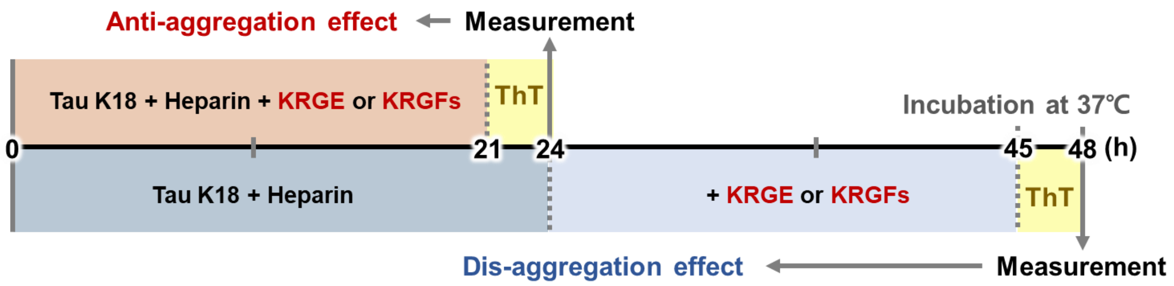

SUPPLEMENTARY FIGURE 4: Overview of the thioflavin T (ThT) assay for evaluating the effect of Korean red ginseng extract (KRGE) and KRGE fractions (KRGFs) on the aggregation and dissociation of tau K18 fragments.

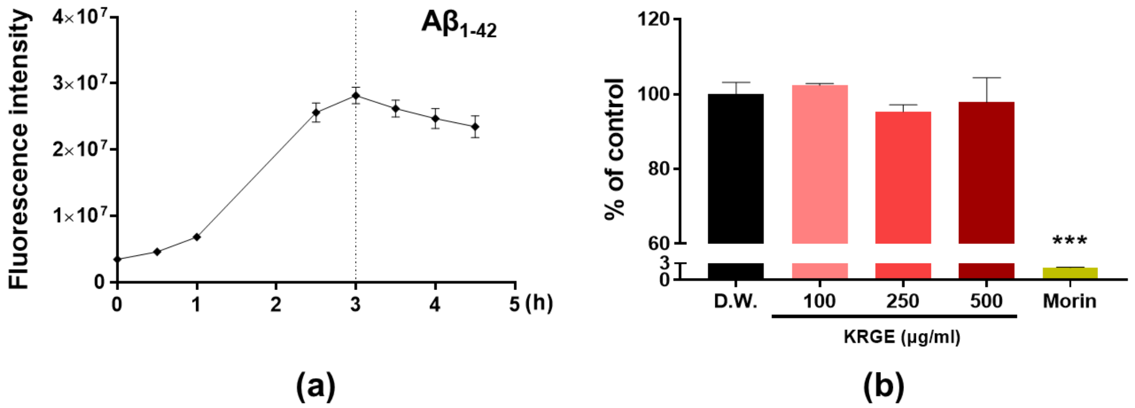

SUPPLEMENTARY FIGURE 5: Evaluation of the effect of Korean red ginseng extract (KRGE) on $A\beta_{1-42}$ aggregation. (a) Fluorescence intensity during the thioflavin T (ThT) assay. Monomeric $A\beta_{1-42}$ was incubated for 5 hours to allow for oligomerization. (b) The ThT assay was performed to examine the effect of KRGE on $A\beta_{1-42}$ aggregation. When $A\beta_{1-42}$ was incubated in the presence of morin, a well-known inhibitor of Aβ aggregation, $A\beta_{1-42}$ aggregation was dramatically reduced. However, no significant change in $A\beta_{1-42}$ aggregation was observed after incubation with KRGE. Values represent the mean ± standard error of the mean of three independent experiments. Statistical significance was determined by one-way analysis of variance followed by Tukey's multiple comparisons test. $^{***}p < 0.001$.
